# Efficacy of low-dose rituximab in minimal change disease and prevention of relapse

**DOI:** 10.1186/s12882-023-03092-7

**Published:** 2023-04-26

**Authors:** Jian Zhang, Hui Zhao, Xiaoli Li, Rui Qian, Peijuan Gao, Shouyan Lu, Zhigang Ma

**Affiliations:** 1 Division of nephrology, Gansu Provincial Hospital, Lanzhou, 730001 China; 2 Department of nephrology, The Second Affiliated Hospital, School of Medcine, The Chinese University of Hong Kong, Longgang District People’s Hospital of Shenzhen, Shenzhen, Guangdong 518172 China

**Keywords:** Minimal change disease (MCD), Rituximab (RTX), Relapse

## Abstract

**Background:**

Minimal change disease (MCD) is a major cause of nephrotic syndrome (NS) in children and a minority of adults. The higher tendency to relapse put patients at risk for prolonged exposure to steroids and other immunosuppressive agents. B cell depletion with rituximab (RTX) may be beneficial to the treatment and prevention of frequently relapsing MCD. Therefore, this study aimed to verify the therapeutic/preventive effects of low-dose RTX on the relapse in adult with MCD.

**Methods:**

A total of 33 adult patients were selected for the study, including 22 patients with relapsing MCD in relapse treatment group who were treated with low-dose RTX (200 mg per week × 4 following by 200 mg every 6 months) and 11 patients in relapse prevention group with complete remission (CR) after steroid therapy were treated with RTX (200 mg ×1 every 6 months) for preventing the relapse of MCD.

**Results:**

Of the 22 patients with MCD in relapse treatment group, there were 21 cases (95.45%) of remission [2 (9.09%) partial remission (PR), 19 (86.36%) CR], 1 (4.56%) no remission (NR) and 20 (90.90%) relapse-free. The Median duration of sustained remission was 16.3 months (3, 23.5 months, inter quartile range (IQR)). 11 patients in the relapse prevention group during a follow-up of 12 months (9–31 months) had no relapse. The average dose of prednisone in two groups after RTX treatment was significantly lower than before treatment.

**Conclusion:**

The results of this study suggested low-dose RTX can significantly reduce relapse rate and steroid dose in adults with MCD with fewer side effects. Low-dose RTX regimens may be beneficial for the treatment of relapsing MCD in adults and may be the preferred regimen for patients at high risk for the development of adverse events from corticosteroids.

## Background

Minimal change disease (MCD) accounts for approximately 10-15% of adults with idiopathic nephrotic syndrome (NS) [1]. MCD is the most common cause of NS, characterized by edema, heavy proteinuria, and hypoalbuminemia [2]. MCD has a high tendency to relapse, although most of the adult patients respond satisfactorily to steroids and immunosuppressive agents, such as calcineurin inhibitors (CNI), cyclophosphamide, and mycophenolate mofetil [8], which can lead to potential side effects such as infection, diabetes, osteoporosis, and obesity [9]. Renal biopsy of MCD shows no obvious glomerular lesion by light microscopy and absence of deposits by immunofluorescent microscopy, or occasionally presence of small amounts of immunoglobulin M (IgM) in the mesangium [3–5]. Previous studies have shown different aspects of T cell regulation and function in driving podocyte injury in MCD [6, 7]. B cells might be involved in the pathogenesis of MCD, and their depletion is associated with response, while their repopulation is related to relapse [10]. B cell-depleting therapies have also been shown effective in many renal diseases caused by the presence of autoantibodies, such as membranous nephropathy, antibody-mediated rejection (AMR) after kidney transplantation, and antineutrophil cytoplasmic antibody (ANCA)-associated vasculitis [12–14]. Selective B lymphocyte depletion with rituximab (RTX) was expected to inhibit the production of autoantibodies involved in the pathogenesis of the disease without the adverse effects (AEs) of nonspecific immunosuppression [15]. In recent years, RTX has been used for the treatment of refractory NS [11], combined with lower doses of steroids and immunosuppressive therapy. However, there are currently many studies on RTX in children with MCD, instead in adults with MCD. In particular, the dosage of RTX, the duration of treatment, and the need of additional drugs in adult with MCD are unknown. Therefore, this study aimed to verify the therapeutic effect of low-dose RTX on the relapse in adult MCD patients. In addition, we aimed to use low-dose RTX to prevent relapse in adult MCD patients who achieved complete remission (CR) via a follow-up evaluation in the study.

## Methods

This retrospective study aimed to observe the long-term efficacy, relapse, and AEs of RTX for MCD. Thirty-three patients (age > 18 years) with biopsy-confirmed MCD from the Department of Nephrology at Gansu Provincial Hospital in China were included in this study. All patients signed the informed consent form before the onset of medication. 22 of 33 patients had relapsing MCD after being treated with prednisone and other immunosuppressive agents, some of whom relapsed more than once. The annual number of relapses before RTX treatment was the total number of relapses divided by the total disease duration (years), and the annual number of relapses after RTX treatment was the number of relapses divided by the follow-up period starting from the first dose of RTX (years). These 22 patients were in the relapse stage before RTX treatment, and they were designated as the relapse treatment group. The remaining 11 MCD patients with multiple relapses were in CR due to corticosteroids treatment prior to RTX, these patients were designed as the relapse prevention group.

## Study patients

The selected patients had an estimated glomerular filtration rate (eGFR) of more than 30 mL/min as calculated by the Chronic Kidney Disease Epidemiology Collaboration (CKD EPI) equation, and had completed at least 1 course of RTX treatment (relapse treatment group, 4 weekly infusions of 200 mg; relapse prevention group, 200 mg each time) at the Gansu Provincial Hospital. All patients were evaluated prior to RTX administration to exclude infectious and non-infectious conditions that contraindicate RTX. Dexamethasone and promethazine were given as a preventive anti-allergic treatment before drug infusion. Additionally, ECG monitoring was performed during infusion, as the most common and serious adverse reaction of RTX is the allergic reaction during the first infusion.

## Study procedures

**Fig. 1:**
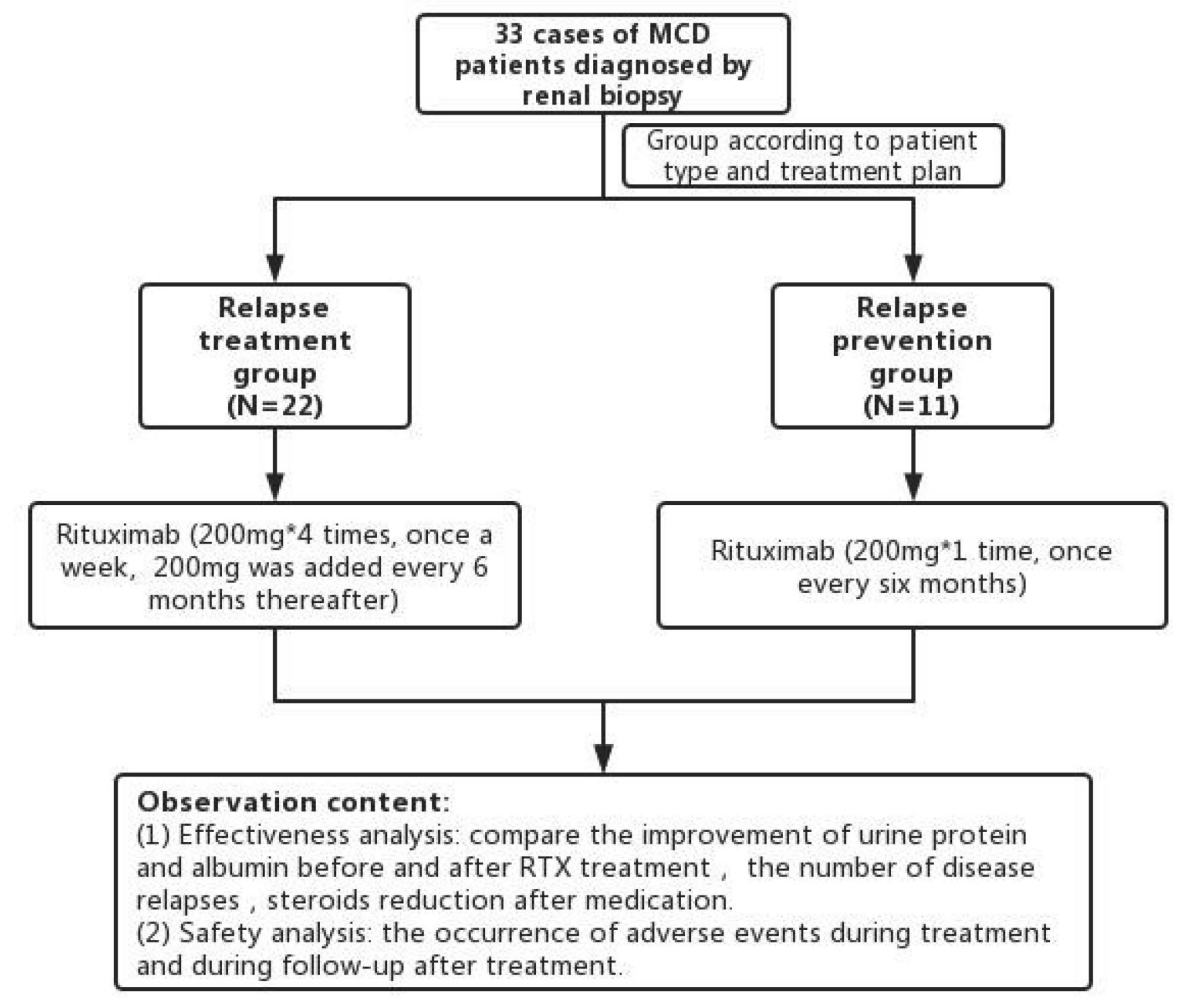
Flow chart of patient

The treatment plan for the relapse treatment group was 4 doses of 200 mg, once a week initially, and maintenance therapy was 200 mg every 6 months. At the same time, we used RTX (200 mg per dose) to prevent relapse in 11 patients in the relapse prevention group, who achieved CR previously and observed the follow-up effect after RTX administration. The research flow chart is shown in Fig. 1. Steroids and immunosuppressants were gradually reduced and ultimately withdrawn after receiving RTX treatment in the remission group, however, oral renin-angiotensin system (RAS) inhibitors were not halted.

Data were collected at baseline, the first month, every 3 months after RTX use, at relapse, and at the last follow-up. The collected data mainly included basic information of patients (such as gender, height, and weight), age of onset, previous immunosuppressive therapy, disease duration, and relapse rate from the medical history. Clinical data were obtained from medical records at admission, including age at the first RTX infusion, sex, comorbidities, current immunosuppressive therapy regimens, and the dosage of steroids before RTX therapy and at last follow-up. Biological data were obtained from laboratory databases, including 24-hour urine protein excretion, serum albumin, CD19 + percentage of B cells, serum creatinine, and eGFR calculated using the CKD-EPI equation.

Remission was defined based on 2012 Kidney Disease Improving Global Outcomes (KDIGO) clinical practice guidelines for glomerulonephritis [16]. CR was defined as the condition with a reduction of proteinuria to 0.3 g/d, normal serum creatinine and serum albumin of > 35 g/L. Partial remission (PR) was defined as a state with a reduction of proteinuria to 0.3–3.5 g/d, a decrease of > 50% from the baseline value, and a stable serum creatinine level (change in serum creatinine of < 25%). Relapse was defined as the recurrence of proteinuria > 3.5 g or UPCR > 3500 after 3 months of remission, and inlcuded episodes after partial response. The study protocol was approved by the medical ethics committee of Gansu Provincial Hospital and the study was performed following the Declaration of Helsinki.

## Statistical analysis

All continuous variables were expressed as mean ± SD or median (interquartile range). The outcome variables evaluated in the study included the number of relapses before and after treatment with RTX and the reduction of prednisone treatment. The numbers of relapses per year before and after treatment were compared using student’s t-test. P < 0.05 was considered statistically significant.

## Results

### Patients’ characteristics

The baseline characteristics of the study population are presented in Table 1. All patients were clearly diagnosed with MCD through renal biopsy and clinical manifestations. The median ages of the relapse treatment group and the relapse prevention group were 32 years (18–60 years) and 25 years (18–58 years) at disease onset. The median age of patients at initiation of RTX was 32.7 years (20.2 to 60.3 years) and 26.7 years (19.3 to 58.6 years), respectively. The time from renal biopsy to initiation of RTX was 10 months (4.0–72.0 months) and 7 months (4.0–20.0 months), respectively. At baseline, all patients in the relapse treatment group had NS, while patients in the relapse prevention group had CR after treatment with corticosteroids agents. All 33 patients received at least 1 course of corticosteroids or other immunosuppressive therapy in the relapse treatment group, such as cyclosporin (n = 1), tacrolimus (n = 1), cyclophosphamide (n = 3), and mycophenolate mofetil (n = 1). The baseline prednisone dosage of the two groups during RTX treatment were 25.0 mg/day (5-60 mg/day) and 10 mg/day (5-30 mg/day). Two of these patients developed secondary diabetes after steroid treatment and then they were treated with tacrolimus and cyclosporine alone. After the treatment with RTX, mycophenolate mofetil and cyclophosphamide were stopped, and the other two patients treated with CNI drugs had CR after one month of RTX treatment, and CNI drugs were stopped.

**Table 1:** Clinical characteristics of study population at baseline

Characteristics	Patients in relapse treatment group (n = 22)	Patients in relapse prevention group (n = 11)
Age years, Median (IQR)		
at disease diagnosis	32 (18–60)	25 (18–58)
age at RTX start	32.7(20.2–60.3)	26.7(19.3–58.6)
Sex ratio (F/M)	7/15	3/8
Blood-Pressure,mmHg, Mean ± SD		
Systolic	123.6 ± 9.8	120.5 ± 10.3
Diastolic	73.6 ± 7.6	74.8 ± 6.8
Height, cm, Mean ± SD	168.8 ± 8.1	166.9 ± 7.9
Weight, Kg, Mean ± SD	66.0 ± 10.7	64.8 ± 15.1
24-hour urinary protein excretion, g, Mean ± SD	8.60 ± 6.10	0.09 ± 0.04
Serum albumin, g/L, Mean ± SD	28.1 ± 9.9	43.2 ± 3.9
Serum creatinine,µmol/L, Mean ± SD	73.2 ± 24.4	63.1 ± 9.8
eGFR (CKD-EPI), ml/min*1.73m2, Mean ± SD	111.5 ± 29.4	127.5 ± 22.4
Time from kidney biopsy to rituximab infusion months, Median (IQR)	10 (4.0–72.0)	7 (4.0–20.0)
Percentage of CD19 + B lymphocytes, %, Median (IQR)	11.6 ± 4.6	8.2 ± 4.6
Previous immunosuppressive therapy		
Long term corticosteroids	22	11
Cyclosporine	1	0
Tacrolimus	1	0
Cyclophosphamide	3	0
Mycophenolate mofetil	1	0
Prednisone dose before rituximab therapy,mg/day, Median (IQR)	25.0 (5–60)	10 (5–30)
Relapse rate before rituximab therapy, number/year, Median (IQR)	1.0 (0.2-3)	0.7 (0–2)
Follow-up time, months, Median (IQR)	19.5 (10–24)	12 (9–31)

Quantitative data were expressed as median (inter quartile range IQR) or mean ± standard deviation (SD), categorical data were presented as frequencies. eGFR, estimated glomerular filtration rate; CKD-EPI, chronic kidney disease epidemiology collaboration

Table 1 also shows the clinical parameters of the two groups before RTX treatment. The baseline 24-hour urine protein level in the relapse treatment group before RTX was 8.60 ± 6.10 g, and the serum albumin was 28.6 g/L (13.9–36.8 g/L). Patients in the relapse prevention group achieved CR with corticosteroids. The baseline eGFR of the two groups was 111.5 ± 29.4 mL/min per 1.73 m^2^ and 127.5 ± 22.4 mL/min per 1.73 m^2^. The percentage of CD19 + B cells in the relapse treatment group and the relapse prevention group before RTX treatment was 11.6%±4.6% and 8.2%±4.6%, respectively. The percentage of CD19 B + cells is the total number of lymphocytes. Due to the limitations of laboratory equipment, we could not obtain an absolute count of CD19 + B cells, but this limitation did not affect our observation of the B-cell depletion effect of RTX. The mean follow-up time after the first infusion was 19.5 months (10–24 months) and 12 months (9 to 31 months) for the other group. The median RTX infusion in the relapse treatment group and the relapse prevention group for the prevention of relapse was 7 (5,7, IQR) and 2 (2,6, IQR).

### Relapse treatment group

**Fig. 2:**
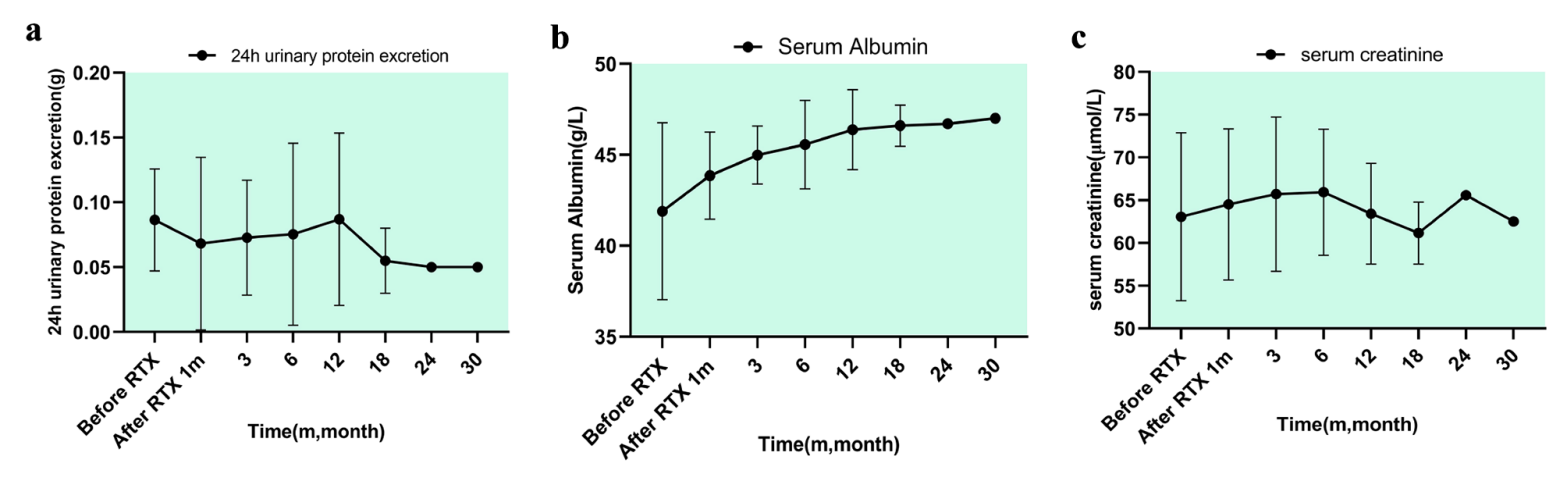
a, Changes in urine protein excretion after RTX therapy (*P* < 0.01); b, Changes of serum albumin after RTX therapy (*P* < 0.01); c, Changes in serum creatinine after RTX therapy. Abbreviations: RTX, Rituximab

The changes in 24-hour urinary protein excretion, serum albumin, and serum creatinine are illustrated in Fig. 2; Table 2. The baseline albumin and 24-hour urine protein quantitative indexes before RTX treatment were statistically significantly different from those at the last follow-up (*P* < 0.001). Of the 22 MCD patients in relapse treatment group, there were 21 cases (95.45%) of remission [2 (9.09%) PR, 19 (86.36%) CR], 1 (4.56%) NR. During the follow-up period following the medication, the renal function of all patients remained stable (shown in Fig. 2C).

**Table 2:** Changes in proteinuria, serum creatinine, and serum albumin after RTX treatment (relapse treatment group)

Clinical parameters	Before RTX	After RTX 1 m	3 m	6 m	9 m	12 m	18 m	24 m
**Proteinuria, g/24 h**	8.60	2.30	1.61	1.48	1.03	0.88	0.80	0.10
**Serum Albumin, g/L**	23.1	36.9	39.1	41.0	42.5	43.2	43.8	43.5
**Serum Creatinine, µmol/L**	73.2	63.9	68.4	66.4	69.3	67.8	66.2	61.3

RTX, rituximab

**Fig. 3:**
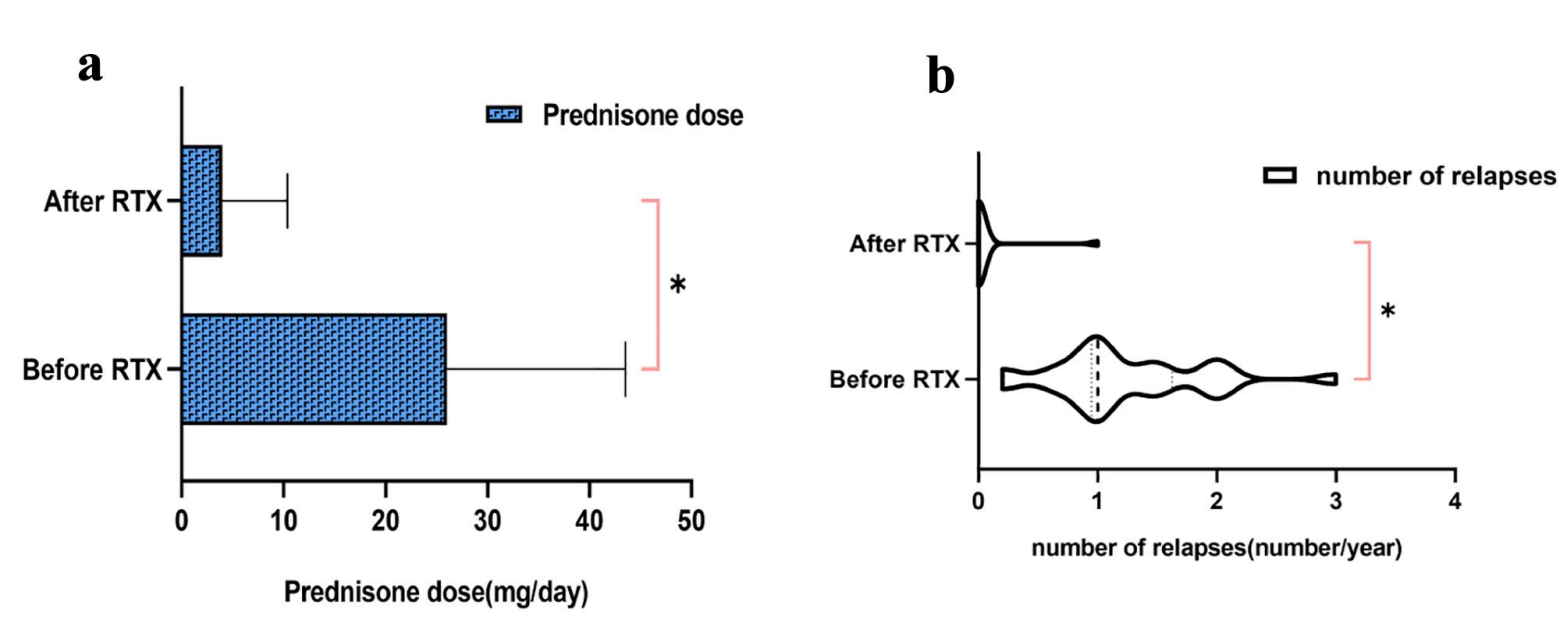
a, Prednisone dose is significantly different in patients of the relapse treatment group before and after RTX treatment (*P* < 0.01). b, The number of relapses ± SD per year before and after RTX treatment (*P* < 0.01). Abbreviations: RTX, rituximab *P*-value was calculated using paired t-test to demonstrate the differences in prednisone dosage and relapse rate between before and after RTX treatments

For the changes in steroid dosage before and after the intervention of RTX, the data at the first administration of RTX and the data at the last follow-up were included for comparison. The mean steroid dose at the last follow-up was significantly lower than before RTX (26.0 mg/d vs. 4.0 mg/d), and the average relapse rate after RTX was significantly lower than before (0.06 vs. 1.24) (*P* < 0.001) (Fig. 3). Of the 21 patients in remission, only one patient in PR relapsed during a brief period of prednisone administration following RTX. The patient achieved PR treated with low-dose RTX (200 mg per week × 4). Six months later, an additional dose of 200 mg was given. At 8 months of follow-up, relapse occurred with increased serum creatinine. We considered the presence of some trigger for relapse. It may be related to the transient diarrhea that occurred at that time. Two patients had steroid-caused secondary diabetes before treatment with RTX, and both patients achieved CR with a brief period of withdrawal from prednisone. After that, the abnormal blood glucose was gradually improved. In addition, we found that thirteen of the nineteen patients who achieved CR stopped the prednisone at the last follow-up, and the remaining patients only took a small dose of prednisone (5-7.5 mg/d). Nineteen patients achieved CR for an average of 6.7 weeks (1,36 weeks, IQR) after the first infusion of RTX. The above data is detailed in Table 3.

**Table 3:** Prednisone dose changes and remission

	CR	PR	NR
**Number of cases**	19	2	1
**Prednisone dose at last follow up, mg/day**	1.7 (0-7.5)	17.5 (15–20)	20
**Remission time, weeks**	6.7 (1–36)	14 (4–24)	/
**Remission maintenance time, months**	16.3 (3-23.5)	9 (5–13)	/
**Number of relapses**	0	1	/

Complete remission, CR; partial remission, PR; no remission, NR

### Relapse prevention group

All patients in relapse prevention group achieved CR with steroid therapy only and no other immunosuppressive therapy, when the steroids dosage was reduced to less than 30 mg, RTX (200 mg, once every 6 months) was given as an infusion to prevent relapse. Simultaneously, we stopped the corticosteroids use and continued to use ARB drugs to protect the renal function.

**Fig. 4:**
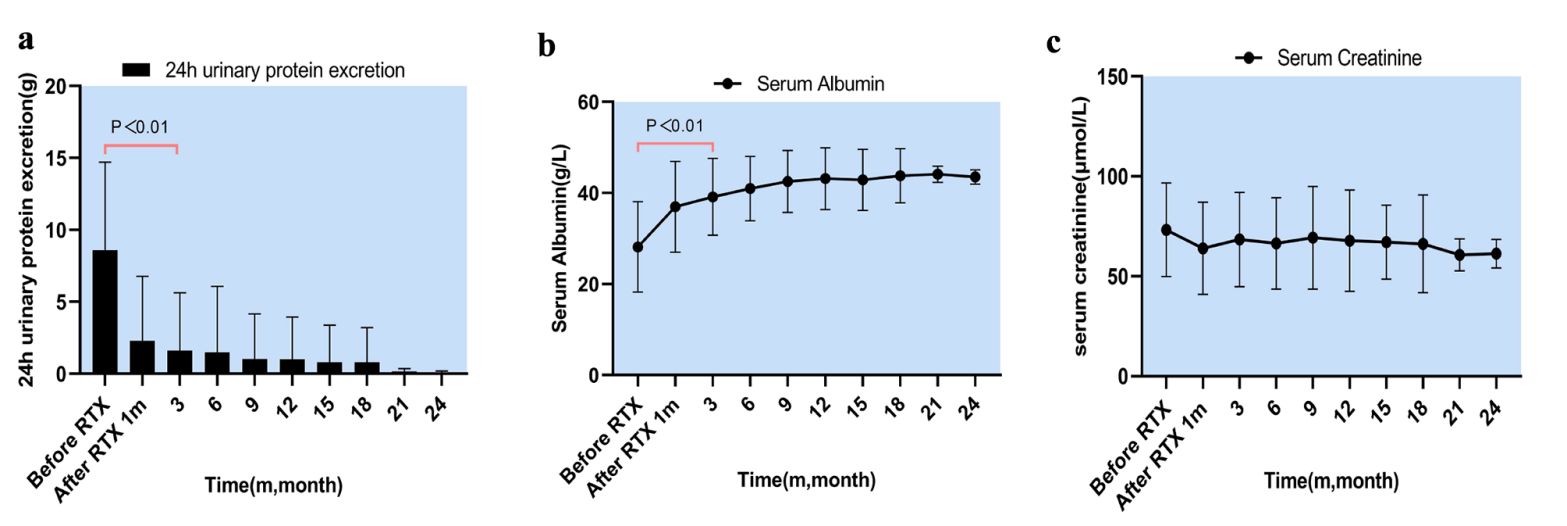
a, Changes in urine protein excretion after RTX therapy; b, Changes in serum albumin after RTX therapy; c, Changes in serum creatinine after RTX therapy Abbreviations: RTX, Rituximab

**Fig. 5:**
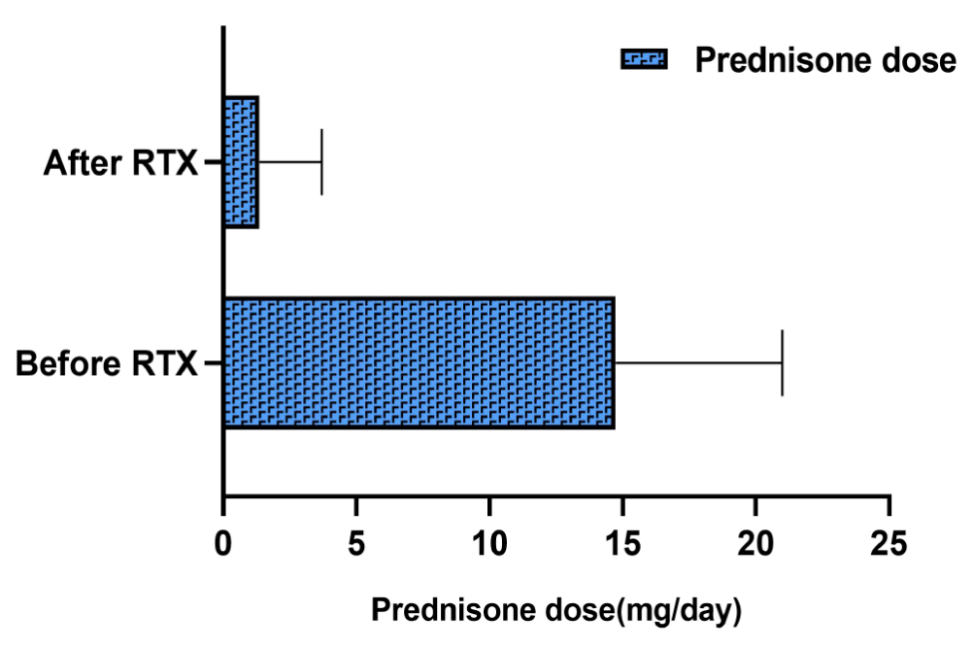
Prednisone dose was significantly different in patients of the relapse prevention group before and after RTX treatment (*P* < 0.01)

Eleven patients were generally in good condition during RTX treatment to prevent relapse and had no AEs. These patients had complete remission of the disease. During the follow-up period of 12 months (9–31 months) after RTX treatment all patients were in a stable condition and had no relapse. The average relapse rate before rituximab therapy was 0.7 (0–2)number/year (IQR). The changes in 24-hour urinary protein excretion, serum albumin, and serum creatinine were illustrated in Fig. 4; Table 4. The mean steroid dose at the last follow-up was significantly lower than before RTX (13.4 mg/d vs. 1.4 mg/d) (*p* < 0.001), which was illustrated in Fig. 5.

**Table 4:** Changes of proteinuria, serum creatinine, and serum albumin after RTX treatment (relapse prevention group)

Clinical parameters	Before RTX	After RTX 1 m	3 m	6 m	12 m	18 m	24 m	30 m
**Proteinuria, g/24 h**	0.09	0.07	0.07	0.08	0.09	0.07	0.05	0.05
**Serum albumin, g/L**	43.2	43.9	45.0	45.6	46.4	46.6	46.7	47
**Serum creatinine, µmol/L**	63.1	64.5	65.7	65.9	63.4	65.9	65.6	62.5

RTX, rituximab

### Changes of the percentage of CD19 B + cells

**Fig. 6:**
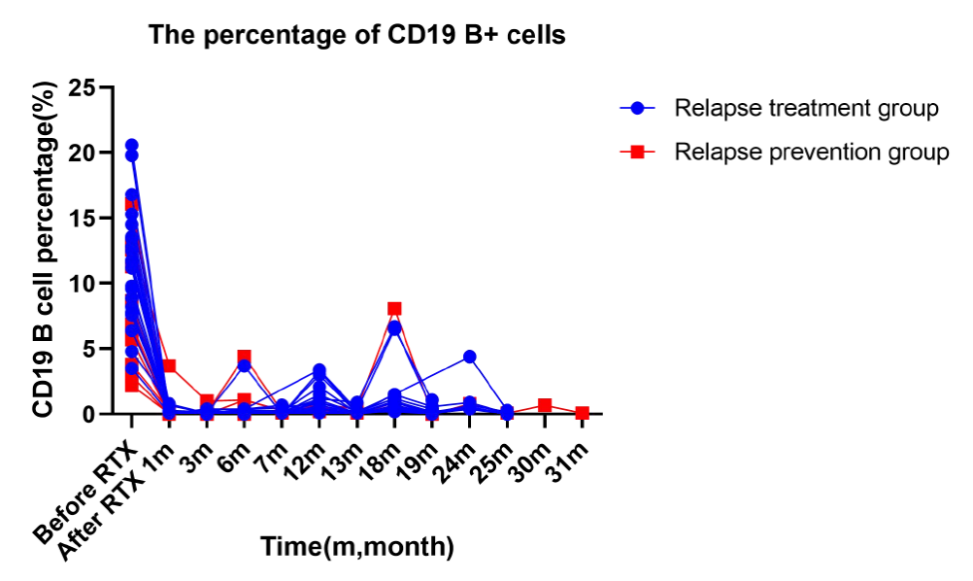
Changes in the percentage of CD19 + B cells in all patients of the two groups before and after RTX treatment

As shown in Fig. 6, both the relapse treatment group and the relapse prevention group treated with RTX, basically completely depleted B cells within a short period of time.

B cells in some patients were gradually increased during a period of 0.5 to 1 year after RTX treatment, consistent with the reported RTX effect lasting about 6–12 months after the onset of action [17, 18].

### AEs

RTX was well tolerated in all patients, and there were no AEs such as infection or hematological toxicity during follow-up. 1 out of 33 patients developed a mild allergic skin rash at the infusion site during the infusion process, which was relieved after symptomatic treatment with dexamethasone and slowing down the infusion rate.

## Discussion

MCD is a major cause of NS in children (approximately 90%) and a minority of adults (approximately 10%). MCD and focal segmental glomerulosclerosis (FSGS) are the main pathogenic manifestations affecting podocytes. For most adults with primary MCD, initial treatment is recommended with glucocorticoid monotherapy. For patients who cannot tolerate, have contraindications to steroid therapy, or do not wish to take high-dose glucocorticoids or glucocorticoid-sparing regimens, there are other options, including calcineurin inhibitors (CNI) or mycophenolate mofetil/enteric-coated mycophenolate sodium (MMF/EC-MPS) plus reduced-dose glucocorticoids. Glucocorticoid monotherapy leads to complete remission in 80 to over 95% of adults with MCD [19–21]. The time course for CR varies, with 50% responding within four weeks and 10 to 25% requiring more than three to four months for treatment. However, disease relapse, glucocorticoid resistance, and drug-related AEs are common issues among adults receiving prolonged high-dose of glucocorticoids as first-line therapy for MCD. Numerous observational studies and a small number of randomized trials have suggested that approximately 70 and 90% of MCD patients, respectively, undergo CR or PR after cyclosporine treatment [22–24]. Similar results have been reported for tacrolimus [25–27]. Over 60% of MCD patients who respond to cyclosporine or tacrolimus will relapse (usually within six months) after stopping CNI if the duration is relatively short [28].

RTX undoubtedly provides us with new strategies for the treatment of MCD, and more and more related studies have emerged recently. Fornoni et al. [29] recently demonstrated that RTX might contribute to stabilize podocyte cytoskeleton and prevent apoptosis through interaction with sphingomyelin phosphodiesterase acid-like 3b protein expressed in glomerular epithelial cells. This observation suggests that RTX therapy may be effective not only through CD20 lymphocyte depletion but also by modulating podocyte function. Observational studies have shown that RTX may benefit adult MCD [30, 31]. In a systematic review and meta-analysis of 21 studies involving 382 adults with FR/GD MCD or focal segmental glomerulosclerosis (FSGS), RTX therapy induced CR in 92% of patients with MCD, although 28% of the patients relapsed during follow-up [32]. RTX was well tolerated and associated with few AEs.

Although RTX has not been recommended as a first-line treatment for MCD and FSGS, it has shown promising results in this study and several other previous studies. The objectives of this study were to actually divide patients into two major groups. In the relapse treatment group, our results demonstrated a remission rate of 95.5% with 86.3% CR and a relapse rate of 0.05 episodes/patients/year. Of the three patients who did not achieve CR, two of them had worse baseline renal function than those with CR. This outcome also suggested that active intervention and treatment in the early stage of the disease will have a better prognosis. Many studies have found most of the patients who did not respond to RTX had advanced kidney impairment, while the renal function was mostly preserved among responders. Kidney dysfunction is associated with treatment resistance and is frequently observed in cases with non-selective proteinuria [33–35].The findings from Allinovi et al. [36] highlighted a strong association between PSI and response to RTX in adults with MCD and FSGS. Proteinuria selectivity Index (PSI), the ratio of urinary immunoglobulin G clearance to transferrin clearance, may help identify patients for whom B-cell targeted therapy is effective. At the same time, we also compared the prednisone dosage before and after treatment. Most patients achieved CR within 3–6 months after the first use of RTX, and the use of prednisone was gradually stopped. This discontinuation greatly reduced steroid-related AEs. The prednisone in the relapse prevention group was also stopped for a short period of time or maintained at only a very small dose.

Even in lymphoma and rheumatoid arthritis, it is difficult to determine the optimal dose of RTX that has the maximum duration of remission and minimal side effects. In our study, the dose of RTX was relatively low with only 200 mg each time, but good therapeutic effects were still achieved. This treatment plan was designed by our clinical center and has been used in the treatment of renal diseases in our center for several years. The 4 weekly RTX infusions of 375 mg/m^2^ treatment mainly considered the application of RTX in hematological diseases, especially non-Hodgkin lymphoma. It is still worth discussing whether B cells need to be completely depleted in immune-related glomerular diseases. At the same time, there were relatively few AEs with low doses. MCD is not associated with autoantibodies in contrast to membranous and lupus nephropathies. It is of interest to try RTX in patients with MCD since a reduced dosage of RTX is able to deplete B cells. A large number of RCT studies are needed to confirm the dose and additional regimen of RTX in renal disease. At present, there are few studies on the use of low-dose RTX in the treatment of MCD internationally. In addition, as an additional benefit of our study, we included patients who achieved CR after corticosteroid and immunosuppressive treatment and were given 200 mg of RTX each time to prevent disease relapse. Eleven patients maintained in CR during an average follow-up period of 12 months. Low-dose and supplemental dose every 6 months made the results reliable and comparable, further facilitating the use and investigation of RTX in MCD patients.

In addition, RTX-related AEs were well tolerated by patients in this study, and no subsequent infections and other negative events occurred. The most common adverse event of RTX was allergic reaction during the first infusion, such as fever, chills, bronchospasm, skin rash, and hypotension, and the incidence of adverse reactions was reduced during the subsequent reinfusion [37, 38]. All patients treated with RTX were given prophylactic antiallergy therapy with dexamethasone and promethazine prior to infusion in our clinical center. In patients with repeated steroid and immunosuppressive therapy, compound trimethoprim was administered to prevent infection after using RTX.

There were some limitations to our study. First of all, the data are sourced from a single-center and a lack of prospective controlled data. Each physician in our department selected RTX therapy for adult MCD patients based on their clinical experience. There was no consensus on patient selection. Moreover, our laboratory only provided percentage of the change in B lymphocyte count, not the absolute value, and these data may have not been able to assess changes more accurately in B cells. Additionally, follow-up time was too short that the relapse rate as well as long-term AEs might have been underestimated. Consequently, investigations including randomized controlled trials with long-term follow up regarding RTX use in adult MCD are required to further determine the effectiveness and safety of RTX.

At present, many clinical research centers are gradually inclined to use RTX to prevent the relapse of MCD [39]. In our study, low-dose RTX was repeatedly given at fixed time schedule of 6 months and not only in B cell repopulation, which is more common in children, and we observed that it also has a good therapeutic effect in adult patients with MCD [40]. RTX may be a good option to prolong remission in adult patients with relapsing MCD.

## Conclusion

The results of this study suggested low-dose RTX significantly reduced the relapse rate and steroid dose in MCD patients. RTX can also relieve proteinuria, contribute to achieve response, and reduce relapse in patients with MCD. RTX which is associated with prolonged remission may have a remarkable efficacy in the treatment ofrelapsing MCD in adults and may be preferred in patients at high risk for the development of AEs from corticosteroids.

## Data Availability

All data generated or analyzed during this study are included in this article. Further enquiries can be directed to the corresponding author.

## Abbreviations

MCD: Minimal change disease.
RTX: rituximab.
AEs: adverse effects.
ANCA: antineutrophil cytoplasmic antibody.
MDRD: Modification of Diet in Renal Disease.
RAS: renin-angiotensin system.
eGFR: estimated glomerular filtration rate.
CR: Complete remission.
PR: Partial remission.
FSGS: focal segmental glomerulosclerosis.
CNI: calcineurin inhibitors.
